# TERRIBLE TRIAD OF THE ELBOW: FUNCTIONAL RESULTS OF SURGICAL TREATMENT

**DOI:** 10.1590/1413-785220172506168821

**Published:** 2017

**Authors:** ROBERTO YUKIO IKEMOTO, JOEL MURACHOVSKY, ROGÉRIO SERPONE BUENO, LUIS GUSTAVO PRATA NASCIMENTO, ADRIANO BORDINI CARMARGO, VITOR ELIAS CORRÊA

**Affiliations:** 1. Shoulder and Elbow Surgery Group, Faculdade Medicina do ABC, Santo André, SP, Brazil.; 2. Department of Orthopedics, Hospital Ipiranga (UGA II), São Paulo, SP, Brazil.

**Keywords:** Elbow joint/physiopathology, Elbow joint/surgery, Joint dislocations, Treatment outcome, Articulação do cotovelo/fisiopatologia, Articulação do cotovelo/cirurgia, Luxações articulares, Resultado do tratamento.

## Abstract

**Objective:**

To evaluate the functional and radiographic results of patients who underwent surgical treatment for terrible triad-type elbow injuries (TTE).

**Methods:**

We retrospectively evaluated 20 patients, including one case with bilateral injuries (total of 21 elbows) that were surgically treated from January 2004 to July 2014. We evaluated the functional results of treatment by measuring the restored range of motion (ROM) of the elbow, using the DASH (Disabilities of the Arm, Shoulder and Hand) and MEPS (Mayo Elbow Performance Score) scores. Complications and the development of osteoarthritis and heterotopic ossification (HO) were also evaluated.

**Results:**

Eight elbows (38%) required additional surgical treatment; HO was observed in eight elbows (38%) and severe osteoarthritis (Broberg-Morrey type IV) was seen in only one case (4%). Nevertheless, we obtained good functional results, 14.27 on the DASH and 84 on the MEPS. The average ROM for flexion-extension was 101° (20-140°) and for pronation-supination was 112.85° (0-180°).

**Conclusion:**

When TTE injuries are treated systematically, even despite variations in these injuries, functional ROM and scores ranging from good to excellent can be obtained. **Level of Evidence IV, Case Series.**

## RESUMO

**Objetivo:**

Avaliar os resultados funcionais e radiográficos dos pacientes que sofreram lesões do tipo tríade terrível do cotovelo (TTC) e foram tratados cirurgicamente.

**Métodos:**

Foram avaliados retrospectivamente 20 pacientes, um caso com lesão bilateral (21 cotovelos), que foram tratados cirurgicamente no período de janeiro de 2004 a julho de 2014. Os resultados funcionais do tratamento foram avaliados pela medida da restauração do arco de movimento (ADM) do cotovelo, de acordo com os escores DASH (Disabilities of the Arm, Shoulder and Hand) e MEPS (Mayo Elbow Performance Score). Além da presença de complicações, avaliou-se osteoartrose e ossificação heterotópica (OH).

**Resultados:**

Oito cotovelos (38%) foram submetidos a novo procedimento cirúrgico; observou-se OH em oito cotovelos (38%) e apenas um caso (4%) de artrose grave (tipo IV de Broberg-Morrey). Apesar disso, foram obtidos bons resultados funcionais, DASH de 14,27 e MEPS de 84. E o ADM médio de flexão-extensão foi de 101^o^ (20^o^ e 140^o^) e de pronação-supinação, 112,85^o^ (0^o^ até 180^o^).

**Conclusão:**

Quando se realiza tratamento sistematizado nas lesões do tipo TTC, mesmo com suas variações, pode-se obter um ADM funcional e escore funcional entre bom e excelente. **Nível de Evidência IV, Série de Casos.**

## INTRODUCTION

Hotchkiss first used the term terrible triad of the elbow (TTE) to describe injuries combining posterior-lateral elbow dislocation with fractures of the radial head and the coronoid process.^1^ The “terrible” denotation comes from the fact that this type of injury historically has been difficult to treat and presents poor functional outcomes, especially when compared to simple cases of elbow dislocation.^1^

The goal in treating these injuries is to restore early elbow stability to avoid complications such as loss of function and joint stiffness.^1^ ^,^ ^2^ Over time, surgery has been shown to be the best option for obtaining satisfactory functional results.^3^ The literature shows differences between the surgical techniques used with regard to access routes and the approach to the affected bone structures and ligaments.^3^ ^,^ ^4^

Despite the difficulty in treating TTE-type injuries, a recent systematic review showed that mean functional scores in current studies for the Disabilities of Arm, Shoulder and Hand (DASH) and Mayo Elbow Performance Score (MEPS) assessments are generally between excellent and good.^5^ ^-^ ^7^

Because this injury is complex and difficult to treat (even though good results may be obtained when complications are present), we conducted an evaluation of the cases we have treated surgically in our service over a 10-year period. We compared whether the protocol we used for treatment, functional outcomes, and complication rates were similar to those described in the literature.

The objective of this study was to conduct a retrospective evaluation of the functional and radiographic outcomes in patients who suffered terrible triad-type injuries to the elbow and were surgically treated from January 2004 to July 2014.

## MATERIALS AND METHODS

We identified 29 patients who suffered TTE-type injuries and underwent surgery from the Shoulder and Elbow Surgery Group at Hospital Mário Covas in Santo André, SP and at the Hospital do Ipiranga in São Paulo, SP from January 2004 to July 2014. The study included patients who had a minimum follow-up time of six months and a maximum follow-up of 10 years. We excluded patients who had associated injuries to the affected elbow or forearm, which could alter functional outcome, such as ipsilateral fractures in the arm and forearm, as well as patients who were skeletally immature. The project was approved by the institutional review board at the Hospital do Ipiranga - SP (UGA II) under process number 1659206, and participants signed a term of free and informed consent.

Twenty patients met the criteria, and one case had bilateral involvement, totaling 21 elbows. Most of the patients (16) were male, and four patients were women. The mean patient age was 38.75 years (18-64). Nineteen patients were right-handed and only one was left-handed. The dominant side was affected in 11 individuals.

The most common trauma mechanism was falls from height, which occurred in 10 patients (50%), followed by falls to the ground in 4 patients (20%). The other mechanisms were motorcycle accidents in 3 patients (15%), falls from skateboards in 2 patients (10%), and falling down the stairs in 1 patient (5%).

**Figure 1:**
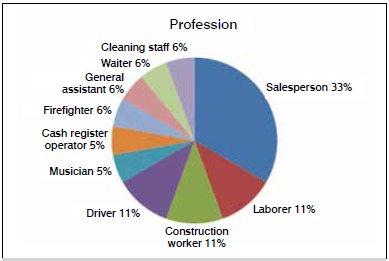
Patient occupations at time of trauma.

The patients’ professions are shown in Figure 1. Of the 20 patients, two were not employed at the time of the trauma. Of the employed patients, 78% took an average of 7.84 months (one month to 18 months) of injury leave, as shown in Table 1.

**Table 1:** Percent of patients who returned to work, and average time of leave.

Return to work		Time (months)
Yes	13 (72%)	7.84
No	5 (28%)	19

Imaging studies, x-rays, and computed tomography scans were used to obtain preoperative classification of the radial head and coronoid fractures. According to the Mason-Johnston classification for radial head fractures, all cases were type IV, associated with dislocation of the elbow.^8^ We also evaluated the number of fragments. Two cases showed only one fragment; 9 elbows (42.8%) had two fragments, 3 cases (14.2%) had three fragments, and 7 elbows (33.3%) had more than 4 fragments. To assess the fractures of the coronoid process, we used the classification proposed by Reagan-Morrey (RM).^9^ Sixteen cases (76%) were type I, 3 (14%) were type II, and 2 (10%) were type III. 

The average time from trauma until surgery was 18.8 days (2-38).

All patients were operated in the dorsal decubitus position. The most common access route was a single lateral access, in 14 elbows (66.67%). To treat the radial head fractures, we used arthroplasty and internal fixation equally (10 cases each), and in only one case the fragment was removed. Three of the 10 arthroplasties were modular-type procedures, and 7 were non-modular procedures. The coronoid process was not approached in 16 patients (76.2%). The lateral ligament complex (LLC) was approached in 20 elbows, while the medial ligament complex (MLC) was not approached in most of the cases, in 16 elbows (76.2%). External articulated fixation was used in 4 patients (19%) due to residual instability. The distal radioulnar joint was treated with provisional stabilization using Kirschner wires in 3 cases (14.3%). Treatment data are summarized in Table 2.

**Table 2:** Treatment.

**Patient**	**Age**	**Sex**	**Via**		**RH**	**TT RH**	**Prot**	**RM**	**TT RM**	**LLC**	**MLC**	**Art Fe**	**DRUJ**	**Other**
**1**	45	M	Dup	LAT-MED	2	Prosthesis	BIP	2	Trans	Trans	Trans	No	No	No
**2**	46	M	Uni	LAT	3	Rafi		1	No	No	No	No	No	No
**3**	33	M	Uni	LAT	4	Prosthesis	UNI	1	No	Anc	No	Yes	Kirsch W	No
**4**	46	M	Uni	LAT	4	Prosthesis	UNI	1	No	Trans	No	No	No	No
**5**	36	M	Dup	LAT-MED	2	Ressec		1	No	Trans	Trans	No	No	No
**6**	64	F	Post	LAT	2	Rafi		1	No	Trans	No	No	No	No
**7**	49	M	Uni	LAT	4	Prosthesis	UNI	1	No	Trans	No	No	Kirsch W	No
**8**	18	F	Uni	LAT	2	Rafi		1	No	Trans	No	No	No	No
**9**	32	F	Uni	LAT	3	Prosthesis	BIP	2	Anc	Trans	No	No	No	No
**10**	28	M	Uni	LAT	4	Prosthesis	BIP	1	No	Trans	No	No	No	No
**11**	32	M	Dup	LAT-MED	2	Prosthesis	UNI	2	Anc	Anc	Trans	No	Kirsch W	No
**12**	48	M	Uni	LAT	4	Prosthesis	UNI	1	No	Anc	No	No	No	No
**13**	55	M	Post	LAT-MED	4	Prosthesis	UNI	3	No	Trans	No	Yes	No	Vasc
**14**	29	M	Uni	LAT	2	Rafi		1	No	Trans	No	No	No	No
**15**	35	M	Uni	LAT	2	Rafi		1	No	Trans	No	No	No	No
**16**	34	M	Uni	LAT	1	Rafi		1	No	Trans	No	No	No	No
**17**	40	M	Uni	LAT	1	Rafi		1	No	Trans	No	No	No	No
**18**	33	M	Dup	LAT-MED	4	Prosthesis	UNI	3	Rafi	Anc	Anc	No	No	No
**19**	45	F	Uni	LAT	3	Rafi		1	No	Anc	No	No	No	No
**20 R**	27	M	Uni	LAT	2	Rafi		1	No	Trans	No	No	No	No
**20 L**			Dup	LAT-MED	2	Rafi		1	Trans	Trans	Trans	No	No	No

Notes: VIA: access route used in surgery; DUP: double access; RH: radial head fracture classification; TT RH: radial head fracture treatment; PROT: type of prosthesis used in cases of radial head arthroplasty was used; UNI: unipolar/non-modular; BIP: bipolar/modular; RM: Reagan-Morrey classification for coronoid fractures; TT RM treatment used for coronoid fractures; LLC: treatment of lateral collateral ligament complex; MLC: treatment of medial collateral ligament complex; ART EF: articulated external fixator; DRUJ: distal radioulnar joint injury; OTHER: other associated injuries. Note: patient 20 had bilateral injury (R: right, L: left).

Postoperative treatment involved the use of an axillary-palmar cast at 90º flexion for an average of 18 days (8-20). After the cast was removed, the patients began physical therapy with exercises at home and outpatient sessions.

Functional performance was assessed using DASH and MEPS scores, and also by assessing the range of motion (ROM) of the elbow on the affected side in comparison with the contralateral limb.^5^ ^,^ ^6^ The Broberg-Morrey scale was used to evaluate postoperative arthrosis, and the physicians also looked for formations of heterotopic ossification (HO) at the interview via anteroposterior and lateral x-rays of the elbow.^10^

The statistical analysis of the data used Fisher’s exact test with a 5% significance level (a=0.05).

## RESULTS

The mean postoperative follow-up period was 31.25 months (8-93). The ROM on the affected side showed an average loss of extension from 21° to -70° (standard deviation [SD] 18^o^), while the average flexion was 123° (90-140°, SD 18.7°). The total average ROM for flexion-extension was 101° (20-140°, SD 33.4°). Mean pronation was 49.7° (-40-90^o^, SD 34.5°), and mean supination was 64.5° (0-90°, SD 26.6°), which consequently produced an average total ROM of 112.85° (0-180^o^, SD 54^o^).

The mean MEPS score was 84 (55-100); 7 patients (35%) were considered excellent, 10 (50%) good, 2 (10%) regular, and only 1 patient (5%) was considered to have poor results. The average DASH score was 14.27 points (0-48.3).

Residual instability was only seen in the physical examination in 7 elbows (33%), but none of these patients were symptomatic. One elbow was positive for the pivot-shift test, 1 was positive for varus stress, and 5 were positive for valgus stress.^1^

The mean ROM, tests for residual instability, and functional evaluation scores are shown in Table 3.

**Table 3:** Results.

	EXT	FLE	MFE	PRO	SUP	MPS	PSH	VRI	VGI	DASH	MEPS	BMR
1	-70	90	20	30	30	60	No	No	No	14.6	85	3
2	-15	135	120	50	70	120	No	No	No	7.5	85	1
3	-30	115	85	20	15	35	No	No	Yes	5	95	1
4	-5	140	135	90	90	180	No	No	No	0	100	1
5	-5	140	135	50	50	100	No	No	No	11.3	100	2
6	-20	90	70	0	0	0	No	No	No	9.1	85	2
7	-30	90	60	-40	40	0	No	No	No	48.3	80	2
8	-20	140	120	90	80	170	No	No	No	0.8	100	2
9	0	140	140	90	90	180	No	No	No	0.8	100	1
10	0	140	140	90	70	130	No	No	No	0	85	2
11	-30	110	80	30	80	110	No	No	No	39	60	2
12	-5	145	140	60	90	150	No	No	No	5.8	100	1
13	-30	120	90	10	80	90	Yes	No	Yes	11.6	75	4
14	-50	120	70	55	75	130	No	No	Yes	30.8	55	3
15	-15	110	95	30	80	110	No	No	No	8.3	80	1
16	-20	120	100	60	70	130	No	No	No	10	85	2
17	-10	140	130	80	80	160	No	No	No	3.33	100	1
18	-40	100	60	40	40	80	No	No	No	31.6	60	1
19	-40	140	100	70	45	115	No	Yes	No	15	80	3
20R	-5	135	130	90	90	180	No	No	Yes	23.5	80	2
20L	-10	130	120	50	90	140	No	No	Yes			3

Notes: EXT: extension; FLE: flexion; MFE: mean flexion-extension; PRO: pronation; SUP: supination; MPS: mean pronation-supination; PSH: pivot-shift test; VRI: varus instability; VGI: valgus instability; DASH: DASH score; MEPS: MEPS score; BMR: Broberg-Morrey classification.

In 16 elbows in which the coronoid process was not approached, better ROM scores than the study average were found, 107° flexion-extension and 112.5º pronation-supination; the functional results for these cases were also better, 11.8 on the DASH and 86.6 for the MEPS. However, there was no significant difference compared to patients who underwent a coronoid approach process (p>0.05 for ROM and functional scores).

A better average ROM was obtained for cases in which the medial ligament complex was not approached (mean 107° flexion-extension and 117° pronation-supination), and better functional scores on the DASH and MEPS (11.23 and 86.5, respectively) compared to cases where this approach was required. In cases requiring the MLC approach, the average ROM was 83° flexion-extension and 98° pronation-supination. The functional scores were 23 for the DASH index and 77 for the MEPS index. Again, there was no statistical difference between the groups (p>0.05 for ROM and functional scores).

As for the presence of osteoarthrosis in the joint, the Broberg-Morrey scores showed 8 type I cases (38%), 8 type II cases (38%), 4 type II cases (19%), and only 1 type IV case (4%).^10^

Eight elbows (38%) showed radiographic signs of HO. According to the classification by Brooker et al.^11^ 7 were type I and only 1 was type II.

Eight elbows (38%) required additional surgical treatment. The average time between the first and second surgery was six months (1-12 months). The reasons were 1 deep infection of the surgical site, 2 cases in which the synthesis material was removed because of pain, 4 cases in which the external fixator required removal, and 1 case of joint release for elbow stiffness.

Complications were observed in 4 patients (19%); 1 case of pseudoarthrosis in the neck of the radial head (in an asymptomatic patient), 1 case of neuropraxia of the posterior interosseous nerve, 1 contralateral fracture of the distal humerus, and 1 case of persistent postoperative paresthesia of the ulnar nerve.

## DISCUSSION

The literature currently demonstrates results generally ranging from good to excellent for surgical treatment of TTE injuries.^1^ ^,^ ^3^ ^,^ ^4^ ^,^ ^7^ ^,^ ^12^ ^-^ ^14^ We obtained mean functional scores of 14.27 points on the DASH and 84 on the MEPS for our patients, with 85% of results classified as excellent or good. This corresponds with the literature, including national studies that resemble our socio-economic reality. (Table 4) The average ROM obtained in our study, 101° flexion-extension and 112.85° pronation-supination, also agrees with the literature (Table 4) and is located within functional ROM of the elbow.^1^

**Table 4:** TTE articles.

	Year	N	FE ROM	PS ROM	DASH	MEPS	HO
Current study		21	101	112.85	14.27	84	8
Chen et al.^7^	2015	12	125	126			
Gonçalves et al.^19^	2014	26	112	133	12	87	
Naoki Miyazaki et al.^20^	2014	15	115	132			
Papatheodoru et al.^16^	2014	14	123	145	14		1
Fitzgibbons et al.^17^	2014	11	112	153	19.7		
Garrigues et al.^15^	2011	40	115		16		
Zeiders et al.^13^	2008	32	100		23		
Forthman et al.^14^	2007	22	117	137			
Pugh et al.^12^	2004	36	112	136		88	

Notes: N: number of patients evaluated; FE ROM: mean flexion-extension range of motion, in degrees; PS ROM: mean pronation-supination range of motion, in degrees; DASH: DASH score; MEPS: MEPS score; HO: patients with heterotopic ossification.

Despite the differences between the protocols for surgical treatment, its primary objective is to provide sufficient stability to begin early mobility and return function to this joint. Most of the protocols recommend fixation or arthroplasty of radial head fractures in association with treatment of the fracture/avulsion of the coronoid process, followed by repair of the LLC through transosseous sutures or the use of anchors.^4^ ^,^ ^12^ ^,^ ^13^ ^,^ ^15^ Cases with residual instability have been treated with an articulated external fixator and/or repair of the MLC.^13.14^

In terms of fractures and avulsions of the coronoid process, the treatment protocols indicate the need to fix or repair these injuries, particularly in cases where large fragments are present (RM type III).^12^ However, Papatheodoru et al.^16^ demonstrated that good results and functional ROM can be obtained in cases where small fragments are present (RM types I and II), without the need for a coronoid process approach. The results obtained in our study corroborate this fact, because even though the coronoid process was not approached in the majority of cases (16 elbows, 76%), these patients had better functional ROM than the average for the study (107° flexion-extension and 112.5° pronation-supination) and also better functional results (11.8 on the DASH and 86.6 on the MEPS), although no statistical difference was found.

The MLC is a key structure in valgus stability in the elbow, but there is no consensus in the surgical protocols on the need to approach this complex during TTE treatment.^12^ ^,^ ^13^ When treatment of the coronoid process or anterior capsule, radial head, and LLC provide enough stability for early mobilization of the elbow, the medial approach and use of external fixation can be avoided.^14^ ^,^ ^17^ In contrast, Toros et al.^18^ demonstrated better flexion-extension and flexion ROM in patients who underwent MLC repair than in those who did not receive this repair. Our study obtained better ROM and higher average functional outcomes in cases where the MLC was not approached, in comparison with cases requiring this approach. Even though this difference was not statistically significant, it may arise from the fact that the lesions had lower trauma energy and less tissue damage, consequently leading to a lower rate of complications.^16^

In a recent systematic review, Chen et al.^7^ showed that the most common complication in TTE which did not require surgical treatment was HO, in 12.5% of cases, followed by ulnohumeral arthrosis in 11.2% of cases. These authors concluded that although the complication rates were high, patients generally obtained satisfactory functional results.^7^ We corroborated this conclusion in our study, because even though there was a 38% rate of HO and reoperation in eight elbows (38%), we obtained functional results which were mostly classified as good or excellent, similar to findings in the national literature. Gonçalves et al.^19^ obtained a total of five complications requiring surgical treatment, and Naoki Miyazaki et al.^20^ presented two cases of neuropraxia of the ulnar nerve and a case of heterotopic ossification with stiffness of the elbow.

We understand that there are limitations in our study. Because it is retrospective in nature, the injury treatment protocol could not be further standardized. Another limitation was the small number in the sample; even though it is similar to others found in the literature, this number hindered the statistical analysis.

There are differences between the protocols used in treating TTE injuries, but even despite these differences, the use of a systematic treatment in the surgical approach ultimately provides good functional results and ROM.^12^ ^,^ ^13^ ^,^ ^17^

## CONCLUSION

Despite the difficulty of treating this injury and the high rates of complications, when systematized treatment is followed to treat TTE-type injuries, even with their variations functional ROM and function scores ranging between good and excellent can be obtained in most cases. We use these protocols in our service, especially with increased understanding of the complexity of this injury and the structures involved. As a result, in the majority of patients we obtained functional results ranging from good to excellent.
